# Protocol for tailored *in vitro* neuronal networks on high-density microelectrode arrays with polydimethylsiloxane microstructures

**DOI:** 10.1016/j.xpro.2026.104349

**Published:** 2026-01-31

**Authors:** Anna-Christina Haeb, Hideaki Yamamoto, Paul Roach, Daniel Merryweather, Yuya Sato, Daniel Tornero, Jordi Soriano

**Affiliations:** 1 Department of Biomedical Sciences, Faculty of Medicine and Health Sciences, Institute of Neurosciences, University of Barcelona, 08036 Barcelona, Spain; 2 Department of Condensed Matter Physics, Faculty of Physics, University of Barcelona, 08028 Barcelona, Spain; 3 Universitat de Barcelona Institute of Complex Systems (UBICS), 08028 Barcelona, Spain; 4 Institut d’Investigacions Biomèdiques August Pi i Sunyer (IDIBAPS), 08036 Barcelona, Spain; 5 Research Institute of Electrical Communication, Tohoku University, Sendai 980-8577, Japan; 6 Department of Chemistry, School of Science, Loughborough University, Leicestershire LE11 3TU, UK; 7 Research Organization for Nano & Life Innovation, Waseda University, Tokyo 162-0041, Japan; 8 Centro de Investigación Biomédica en Red sobre Enfermedades Neurodegenerativas (CIBERNED), 28029 Madrid, Spain

**Keywords:** biophysics, neuroscience, biotechnology and bioengineering, material sciences

## Abstract

Complementary metal-oxide-semiconductor (CMOS)-based high-density microelectrode arrays (HD-MEAs) enable neuronal recordings with high spatiotemporal resolution. However, integrating polydimethylsiloxane (PDMS) microstructures onto HD-MEA surfaces to control network architecture is currently challenging and platform specific. Here, we present a protocol for PDMS fabrication, HD-MEA chip preparation, PDMS-HD-MEA microstructure alignment, and cell culture, including alternatives. Our results show reproducible formation of modular networks with characteristic activity patterns across different systems. This protocol supports engineering of defined neuronal architectures while maintaining compatibility with various HD-MEA systems.

For complete details on the use and execution of this protocol, please refer to Sato et al.^1^

## Highlights

• Protocol to generate PDMS-based modular networks for different HD-MEA chip manufacturers
• Steps for PDMS fabrication, coating, and attachment onto HD-MEA surfaces
• Handling and cleaning protocols to reuse and maximize the lifespan of HD-MEA chips
• Details on optimization of the protocol, limitations, and possible solutions

## Abstract (editor highlights)

Publisher’s note: Undertaking any experimental protocol requires adherence to local institutional guidelines for laboratory safety and ethics.

## Graphical abstract

**Figure:**
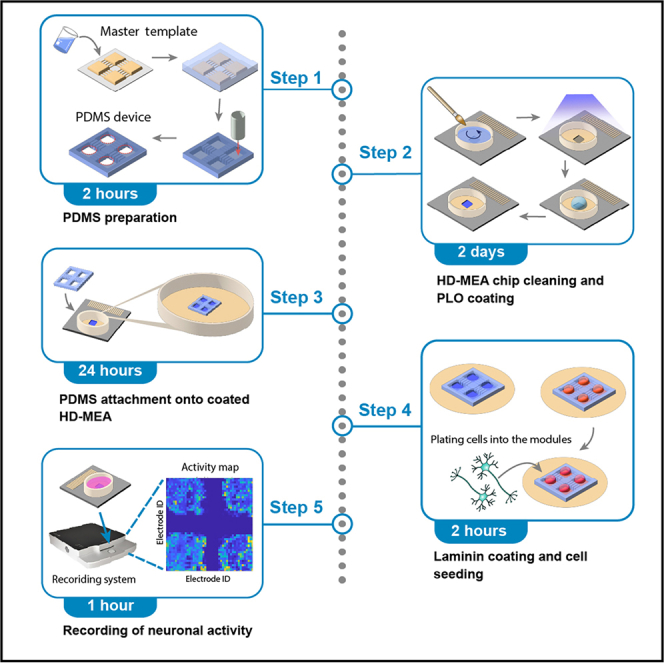

## Before you begin

*In vitro* neuronal networks provide a versatile model for studying neuronal dynamics in controlled and manipulable environments, with applications in drug screening, disease modeling, and information processing.^2^ HD-MEA systems are particularly powerful platforms, enabling monitoring of neuronal activity of the whole neuronal network with high spatiotemporal resolution.^3^

*In vitro* networks are typically grown on flat surfaces, where neurons connect randomly and form highly interconnected structures^4^ that contrast to the highly organized and complex architecture of the brain.^5^^,^^6^ To emulate this brain-like organization, bioengineered microstructures, such as PDMS, can be used to create modular neuronal networks with customized connectivity.^7^ These microstructures, known as brain-on-a-chip, can be integrated onto a MEA system to form advanced neuronal platforms.^8^ However, most existing protocols focus on PDMS with low-density MEAs^7^^,^^9^^,^^10^^,^^11^^,^^12^ rather than on high density ones.^1^^,^^13^ Furthermore, HD-MEA systems vary in chip design, materials, and layout, complicating the development of universal protocols.^14^ Modern HD-MEA systems include those commercialized by 3Brain AG,^15^ Maxwell Biosystems^16^^,^^17^ and MultiChannel Systems.^18^

Before HD-MEA technology emerged, microfabricated PDMS devices were effectively used to control and guide the adhesion sites and axonal growth of cultured neuronal networks on MEAs.^19^^,^^20^^,^^21^ A key advantage of HD-MEAs is their high spatial resolution, in the range 20–50 μm, comparable to the characteristic feature dimensions of PDMS devices.^22^ However, HD-MEA surfaces typically incorporate micrometer-scale 3D topography due to the underlying complementary metal-oxide-semiconductor (CMOS) microcircuitry, which hinders uniform PDMS attachment through simple placement. Attempts to use cushion layers such as hydrogels^1^ or diluted PDMS^13^ may compromise chip reusability, leaving the field without a reliable protocol for attaching PDMS to HD-MEA surfaces.

### Innovation

Given the sensitive nature, high cost, and intended reusability of HD-MEA chips, optimizing their handling is crucial. Therefore, our aim is to provide an adaptable and reproducible protocol with alternatives and general guidelines to: (i) design and prepare PDMS microstructures (‘microfabricated devices’) tailored to specific HD-MEA chips, (ii) integrate these microstructures onto the HD-MEA surface, and (iii) implement cleaning procedures that extend chip lifespan and enhance reusability.

To ensure broad applicability across HD-MEA platforms with different topographies, the protocol is demonstrated on HD-MEA chips with lowered embedded electrodes (3Brain AG). Representative results from a different system featuring raised electrodes (Maxwell Biosystems) are also presented (see Sato et al.^1^ for details).

### Institutional permissions

The preparation of primary neuronal cultures involving the dissections of rat embryonic tissue was carried out in accordance with the regulations of the Ethical Committee for Animal Experimentation of the University of Barcelona (approved ethical order B-RP-094/15–7125 of July 10th, 2015) and the laws for animal experimentation of the Generalitat de Catalunya (Catalonia, Spain). All procedures involving animal experiments at Tohoku University were reviewed and approved by the Center for Laboratory Animal Research, Tohoku University.

### Microfabricated device design

**Timing: Variable depending on design complexity. Usually ∼1 h.**

There are several approaches to performing photolithography to produce a microfabricated PDMS device (often termed microfluidic device, although no fluid flow is involved in this study). The present protocol outlines a general methodology as an introduction to PDMS microfabrication. In a short overview, a ‘master’ inverse template is prepared to serve as a mold for casting PDMS and forming a microfabricated chambered device.^23^ Therefore, a mask aligner or a direct-writing laser system is used. A mask aligner uses a prefabricated photomask, typically consisting of a high-resolution chromium-on-glass pattern, to project UV light onto a photosensitive resin, thereby transferring the pattern. Alternatively, a laser-based direct-writing system can create the pattern directly without the need for a physical mask. Both approaches require a computer-aided design (CAD) of the pattern, either to fabricate the mask or to guide the laser. Direct writing enables much more rapid redesign, without the need to fabricate a new mask for each design change; however, actual printing time is significantly increased. Masked fabrication methods are able to produce a large number of master mold patterns rapidly, but redesigning the mold requires the fabrication of a new photomask.

**Figure 1:**
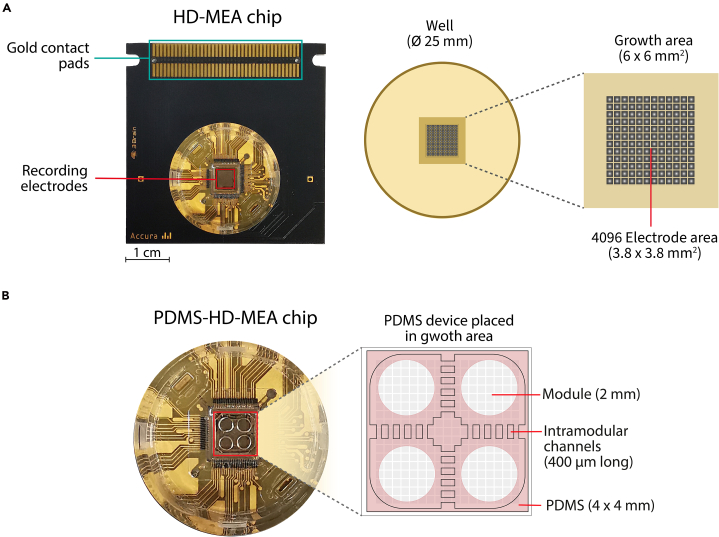
Representative HD-MEA chip from 3Brain together with a PDMS device (A) Standard chip, containing the culture well area where the CMOS sensor sits, together with contact pads to connect to the recording system. The detail of the culture well depicts the 64 ×64 grid of electrodes that records the membrane field potential of the cells growing on its surface. (B) Chip with the PDMS cast sitting on top of the electrodes area, ready for cell culturing. The PDMS structure consists of 4 punched cavities (modules) to place the cells for neuronal culturing. Module pairs are connected orthogonally via a set of 5 parallel channels 400 μm long.

Device design must account for the HD-MEA layout, which includes electrode bed dimensions, electrode positioning within cell chambers and microchannels, and overall chip architecture. The latter is a necessary consideration, with many different types presenting the electrodes quite differently. Some are classed as high-density electro beds (such as 3Brain’s CMOS HD-MEA; Figure 1) having quite tightly packed electrodes, whereas others are much more spread across the HD-MEA surface, sometimes even grouped into specific regions on the HD-MEA surface (e.g., Med64). The electrodes themselves can also be presented quite differently, having relatively flat surfaces, pseudo-flat surfaces (generally discussed as flat but having ∼1–2 μm dips at the electrode tip due to the insulating layer), or presenting 3D-like electrodes where the tip is extended into the z-plane allowing depth of sensing, e.g., into an organoid. Electrode coatings can also pose a consideration, with platinum, or carbon nanotube coated electrodes being relatively robust chemically, yet becoming more easily contaminated, and possibly broken when coming into direct physical contact with a PDMS mounted device. This is especially relevant for HD-MEA reuse, but careful design can help to align the PDMS device chambers where intended, avoiding unnecessary electrode coverage.

**CRITICAL:** The PDMS device should be designed to enable good contact with the surface of the electrode bed for adhesion, avoiding direct contact with electrode tips whenever possible. This promotes stable attachment and supports HD-MEA reusability.

### Computer-aided design

**Timing: Variable depending on design complexity. Usually ∼2 h.**

1. Select the software that best meets your design requirements.

***Note:*** Many CAD software tools are available, either with commercial license or free, e.g. AutoCAD or Fusion 360 (which offers a free educational license), FreeCAD, TinkerCAD, SketchUp, and KLayout, among others.

***Note:*** Consider the design goals, i.e., while 2D drawing is typically required for CAD, many of these tools also support 3D modeling, which can be useful for visualizing and explaining the designs.

2. Create the CAD layout as a 2D design, typically within a single layer.

***Note:*** Free resources are available to give guidance through the design process.

***Note:*** Ensure that the output file format is compatible with downstream applications. Commonly used and flexible formats include.dwg or.dfx; however, some lithography systems require.gds files. Use software tools such as KLayout to import.dxf files and export them in.gds format if needed.

**CRITICAL:** The full pipeline of production must be evaluated at the start to ensure that the correct output file and design meet the requirements of the fabrication process and instrumentation.

**CRITICAL:** The units set in the design file should be set correctly.

3. Once the CAD file is prepared, proceed with the fabrication of the master inverse device.

### Master inverse device generation

**Timing: Variable depending on design and lithography system used. Usually,** ∼2–4 **h.**

Designs can be fabricated on a range of substrates, with silicon wafers commonly used due to their molecularly flat nature. To transfer the CAD designs into three-dimensional molds, a photopolymer (photoresist) is coated onto the substrate. Although multiple photoresists are suitable, this protocol outlines the use of SU-8, an epoxy-based negative photoresist that polymerizes upon exposure to 365 nm UV light followed by thermal curing. The SU-8 selection chosen depends on the height of patterns needed.^24^ Because PDMS devices typically feature microchannels, e.g., to connect neurons present in different chambers, a choice of 100 μm height is selected when cell migration is intended across chambers, whereas much smaller heights on the order of 10 μm is chosen when cell soma need to be restricted while allowing neurite extension through the microchannels.

4. Substrate Preparation:
  a. Clean an appropriate substrate (silicon wafer) with acetone, isopropanol (IPA), and deionized water (dH_2_O).
  b. Dry wafer thoroughly with a stream of dry air or nitrogen.
  c. Apply an oxygen plasma treatment for 30 s at 0.2–0.5 mbar, 30–50 W to ensure cleanliness of the substrate and improve adhesion of the photoresist.
  d. Apply heating to ensure that the substrate is completely dry prior to spin coating the photoresist. As a general guide, bake the substrate on a hotplate at ≳120°C for 5–10 min immediately before spinning.
5. Photoresist Coating:
  a. If heating was performed in the previous step, the substrate needs to be cooled to 20°C–25°C before proceeding.
  b. Dispense sufficient SU-8 to fully cover the substrate. Choose the SU-8 photoresist according to desired feature height. To achieve a height of 10–50 μm use SU-8 3050, and for a height of 100 μm, use SU-8 2100.
    ***Note:*** The heights are selected according to the biological function of the final PDMS device: ∼100 μm for chambers supporting cell migration, and ∼10 μm when restricting cell soma but permitting neurite extension. Only the microchannel height critically affects functionality; overall chamber height simply accommodates cells and medium.
    ***Note:*** Resin grades were selected to achieve the target thickness range without excessive edge-bead or film instability.
    ***Alternatives:*** SU-8 50 (higher viscosity) provides uniform thick films (50–100 μm),^25^ whilst SU-8 10 yields thinner, more controlled layers for thin films.
  c. Spin the coating at the appropriate speed:
    i. SU-8 3050 resin: first 10 s at 500 rpm and 100 rpm/s acceleration, then 30 s at 3,300 rpm and 300 rpm/s.
    ii. SU-8 2100 resin: first 10 s at 500 rpm and 100 rpm/s, then 30 s at 3,100 rpm and 300 rpm/s.
      **CRITICAL:** The height of the resist can differ dramatically depending on the temperature and relative humidity (RH), typically of the laboratory in which the procedure is carried out, and the age/storage conditions of the photoresist. Therefore, the film thickness must be locally validated through calibration (e.g., microscopy or profilometry) because results vary with equipment and oven uniformity, in line with manufacturer guidance.
      ***Note:*** Compensation of the environmental and equipment variability can be achieved by maintaining 20°C–25°C, <40% RH, and adjusting spin speed as needed in relation to local calibration. Calibration should be done in each laboratory as per manufacturers’ recommendations, with range estimates provided. Small changes in viscosity would be made by temperature and humidity, giving rise to changes in thickness at various spin speeds.
6. Soft Bake (Pre-Exposure Bake):
  a. Place the silicon wafer coated with the photoresist resin on a levelled hotplate.
  b. For the SU-8 3050 resin, bake it for 1 min at 65°C, followed by 20 min at 95°C. For the SU-8 2100 resin, bake it for 5 min at 65°C, followed by 20 min at 95°C.
  c. Cool down to 20°C −25°C slowly (∼5°C/min maximum rate).
7. UV Exposure:
  a. Using a mask.
    i. Place a high-resolution photomask (acetate film with the patterns) in contact with the coated wafer.
    ii. Align if necessary, using a mask aligner or contact exposure setup.
      **CRITICAL:** The smaller the feature, the more critical it is for a tight seal between the photoresist and the photomask. This reduces the impact of refraction and pattern blurring and is required for features <5 μm.
  b. Using maskless lithography.
    i. Place the substrate within the instrument and align it.
    ii. Load in CAD file and ensure the sample writing area matches the placement of the substrate.
  c. Expose with UV light (365 nm wavelength) at an appropriate dose using the ML3 instrument fitted with a 385 nm laser. For SU-8 3050 resin use 250 mJ/cm^2^ and for SU-8 2100 use 240 mJ/cm^2^.
    ***Note:*** UV exposure dose varies with lamp type and age, therefore it is important to perform a dose-response calibration to determine the correct exposure energy for each thickness.
8. Post-Exposure Bake (PEB):
  a. Place the wafer on the hotplate facing up.
  b. Bake at 65°C for 1 min.
  c. Increase to 95°C and bake for 5–10 min.
  d. A further increase to ∼115°C can be useful to ensure solvent is removed and to drive full polymerization of the SU-8; this can assist with securing attachment of SU-8 to the surface.
  e. Allow to cool down to 20°C–25°C as slowly as possible to avoid internal stress and photoresist fracture.
9. Development:
  a. Immerse the wafer in SU-8 developer (e.g., Propylene Glycol Monomethyl Ether Acetate, PGMEA) for 5–10 min, or until unexposed SU-8 is removed.
  b. Confirm complete development, therefore submerge the wafer in a non-solvent such as IPA; the presence of unexposed SU-8 will be indicated by the formation of a white precipitate.
  c. Resubmerge in developer until no precipitate forms upon testing.
  d. Gently agitate the solution during development.
  e. Rinse the wafer with fresh IPA.
  f. Dry using a nitrogen gas stream.
10. Hard Bake (optional, but strongly recommended to enhance durability during PDMS casting):
  a. Bake wafer at 150°C for 10–30 min.
  b. Cool down slowly to 20°C–25°C as slowly as possible.
11. Master Inspection and Chemical Modification:
  a. Examine the features for defects using an optical microscope.
  b. Treat the master silicon/SU-8 template with silane to prevent PDMS adhesion by forming a hydrophobic coating.
    ***Note:*** This coating chemically bonds to the surface of both silicon (via its native silicon oxide layer) and SU-8 through silane linkages. Pre-cleaning with an oxygen plasma is recommended to remove contamination and enhance activation, ensuring a clean and effective conformal silane modification.
  c. Add ∼50 μL of hexamethyldisilazane (HMDS) adjacent to the template and reduce the pressure to volatilize the HMDS.
    **CRITICAL:** Silane and the chemical processes associated with it are hazardous and require strict safety precautions. Always work under a chemical fume hood and consult the Safety Data Sheet (SDS) to ensure proper manipulation.
  d. Allow the master to sit for at least 3 h to complete HMDS surface modification.
  e. Store the master template in a clean, dust-free container prior to PDMS casting.

## Key resources table

**Table:** 

REAGENT or RESOURCE	SOURCE	IDENTIFIER
**Biological samples**		

Cortical tissue from healthy E18 rat cortex tissue (CD Rat) Sprague-Dawley rat (*Rattus norvegicus*)	Charles River	N/A

**Chemicals, peptides, and recombinant proteins**		

Acetone (99.8%)	Thermo Fisher Scientific	268310025
DPBS	Sigma-Aldrich	D8537
Extran	Sigma-Aldrich	1075532500
Hexamethyldisilazane	Thermo Fisher Scientific	TS-84769
Isopropanol	Thermo Fisher Scientific	149320250
Laminin (human)	Biolamina	LN411-02
Laminin (mouse)	Sigma-Aldrich	L2020
NeuroFluo NeuO	STEMCELL Technologies	01801
Poly-L-ornithine (PLO)	Sigma-Aldrich	P4957
Propylene glycol monomethyl ether acetate	Sigma-Aldrich	484431
Silicone RTV Encapsulant-QSIL 216 (PDMS alternative)	CHT	1667371
SU-8 2100 resin	Kayaku Advanced Materials, Inc.	Y111075
SU-8 3050 resin	Kayaku Advanced Materials, Inc.	Y311075
SU-8 developer	Kayaku Advanced Materials, Inc.	Y020100
SYLGARD 184 Silicone Elastomer Base	DWO Europe	01673921
SYLGARD 184 Silicone Elastomer Curing Agent	DWO Europe	01673921
Tergazyme	Sigma-Aldrich	Z273287
Trichloro(1H,1H,2H,2H-perfluorooctyl)silane	Sigma-Aldrich	448931

**Experimental models: Organisms/strains**		

Sprague-Dawley rat (*Rattus norvegicus*) (embryonic brain cortices at age 18, mixed gender)	Charles River	CD rat

**Software and algorithms**		

AutoCAD	Autodesk Inc.	https://www.autodesk.com/
BrainWave version 5.5	3 Brain AG	https://www.3brain.com/
MaxLab Live	MaxWell Biosystems AG	https://www.mxwbio.com/

**Other**		

Acetate mask printing	JD Photo Data	https://www.jd-photodata.co.uk/
Acetatemask	JD Photo Data	N/A
Biopsy punchers	KaiMedical	BP-60F
CorePlate HD-MEA chips (3Brain)	3Brain	CorePlate™ 1W 38/60
MaxOne HD-MEA chips (MaxWell)	MaxWell Biosystems	MaxOne Chips
HD-MEA recording system (3Brain)	3Brain AG	BioCAM DubleX 2019
HD-MEA recording system (MaxWell)	MaxWell Biosystems	MaxOne
Heratherm oven	Thermo Fisher Scientific	Heratherm OMH100
JP Selecta Plactronic hotplate	Fisher Scientific	12022385
Mask aligner	SÜSS Microtec	MJB4
Micro-positioning punch system	CorSolutions	PDMS Port Creator
ML3 fitted with a 385nm laser	Durham Magneto Optics	Optics ML3
Si wafer of 4″ CZ Si, N-type (phosphorous), 525 μm thick, 100 oriented, 1–10 Ωcm	Siegert Wafer GmbH	N/A
Spin coater	Laurell Tech	WS-650-23
Plasma cleaner	Harrick	PCD-002-CE
Vacuum chamber, Buerkle Plastic Desiccator	Thermo Fisher Scientific	10182391

## Materials and equipment

• Laminin:
  ○ Thaw laminin at 4°C and aliquot 10 μL on ice. Store it for 3–6 months at −20°C.
• Poly-L-ornithine:
  ○ Store up to 12 months at 4°C.
• SYLGRAD 184 Elastomer kit (base and curing agent):
  ○ Store the Elastomer kit at 20°C–25°C until the indicated expiry date.

***Alternatives:*** We recommend the QSIL 216 PDMS kit, provided by CHT Silicones (Germany).

• SU-8 Resin:
  ○ Store it at 20°C–25°C until the indicated expiry date.
• Silane:
  ○ Store it in a dry and well-ventilated area in a tightly closed, chemically resistant container, away from incompatible materials.
• Acetone:
  ○ Store it in a dry and well-ventilated area in a tightly closed, chemically resistant container, away from incompatible materials.
• Isopropanol:
  ○ Store it in a dry and well-ventilated area in a tightly closed, chemically resistant container, away from incompatible materials.
• PGMEA:
  ○ Store it in a dry and well-ventilated area in a tightly closed, chemically resistant container, away from incompatible materials.
• HMDS:
  ○ Store it in a dry and well-ventilated area in a tightly closed, chemically resistant container, away from incompatible materials.
• PLO:
  ○ Store up until the indicated expiry date at 4°C.

***Alternatives:*** Polyethylenimine (PEI), store until the indicated expiry date at −80°C.

• Extran 5%:
  ○ Prepare 1 L of 5% Extran, diluted in water. Store at 20°C–25°C until the indicated expiry date.
• Tergazyme 1%:
  ○ Store dry at 4°C. For usage, prepare fresh 1% tergazyme solution in sterile dH_2_O.

**Table:** PDMS composition (10:1 weight ratio of base to curing agent)

Reagent	Final concentration
Silicone Elastomer Base	91%
Silicone Elastomer Agent	9%

**Table:** Overview of specific models with the recommended coating and their properties∗

Cell model	1. Coating	2. Coating
Primary derived cultures	PLO (50 μg/mL)	Mouse Laminin (10 μg/mL)
hiPSC-derived cultures^26^	PLO (50 μg/mL) or PEI (100 μg/mL)	Human Laminin (20 μg/mL)

∗Depending on the cell type and differentiation stage, specific coatings are often required to promote cell attachment, growth, and maturation. While primary cortical cultures generated from embryonic day 18 (E18) rat brains require 2 h for preparation and typically exhibit initial neuronal activity within 5 days, human induced pluripotent stem cell (hiPSC)-derived neuronal cultures require about 2 weeks for generation, followed by approximately 3 additional weeks of maturation before the first activity is observed.^4^

In this protocol, we present results obtained from primary cortical neuronal cultures from E18 rat embryos.

## Step-by-step method details

The protocol describes the generation and sterilization of PDMS microstructures, the preparation and cleaning of HD-MEA chips, and the subsequent steps for cultivating a neuronal network within a specific, custom-designed microstructure composed of four interconnected chambers. Particular attention is given to the precise alignment and placement of the PDMS structure atop HD-MEA chip surface, as well as to the coating and cell seeding procedures. A final cleaning protocol is included to enable reuse of HD-MEA chips after experiments. Figure 1A provides an annotated illustration of the HD-MEA chip, highlighting key structural regions referenced throughout the protocol. Figure 1B depicts the fully assembled PDMS-HD-MEA device, characterized by 4 chambers (referred as modules) that orthogonally interconnect through microchannels.

### PDMS preparation and cleaning

**Timing:∼3–4h**

To minimize the risk of contamination, all subsequent steps should be carried out in a sterile environment. Due to the adhesive nature of PDMS, it is also advisable to use sterile, disposable material such as plastic Petri dishes and Pasteur pipettes throughout the procedure (problem 1).

1. Mix the PDMS curing agent and elastomer base in a ratio of 1:10. Begin by pouring the elastomer base into a sterile, disposable plastic Petri dish (Ø 35 mm), and then add the curing agent.

***Note:*** Use an analytical balance for accurate measurement. Since both components are highly viscous, we recommend cutting the tip of the plastic Pasteur pipette to create a wider opening.

***Note:*** The volume of PDMS and the size of the Petri dish will need to be adjusted according to the dimensions of the fabricated master mold.

***Note:*** Uncured PDMS is difficult to clean, therefore, we recommend using a disposable plastic Petri dish as a mixing vessel, as it is clean and dust-free from its packaging and can be discarded after use. However, the preparation may also be carried out in any other sterile container.

**Figure 2:**
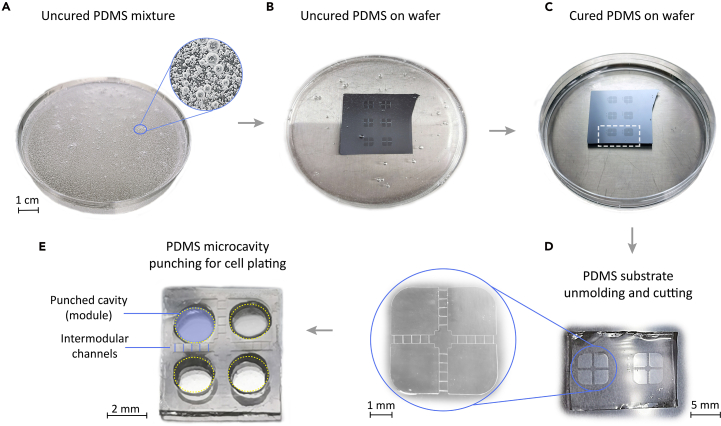
Step-by-step PDMS structure preparation (A) Uncured PDMS mixture. (B) Liquid PDMS poured on top of the resin master wafer, which sits at the bottom of a Petri dish. (C) Cured PDMS. (D) Detail of two PDMS devices ready for unmolding from the master and that correspond to the patterns shown with a white dashed outline in (C). Magnified view of the left device (encircled in blue), showing the details of the crossing channels between module pairs. (E) PDMS cast after unmolding, depicting the 4 punched microcavities that shape the modules where neurons will be seeded.

2. Mix the two PDMS components homogenously thoroughly for 3–5 min until the mixture contains uniformly spread bubbles and all trapped air is released (Figure 2A).

**CRITICAL:** Incomplete mixing of PDMS’ components, especially at the bottom or at the walls of the container, can result in curing agent that may later remain uncured or adhere to surfaces.

3. Use a vacuum chamber to apply a pressure of ∼60 Torr for 15 min to degas the PDMS mixture.
4. Place the device template in a new, sterile plastic Petri dish that fits the dimensions as the master mold. Next, pour the PDMS mixture on top of the mold (Figure 2B).

**CRITICAL:** The volume of PDMS mixture poured will determine the final height of the PDMS structure. A thickness in the range 0.5–1 mm is recommended. Excessive thickness may cause detachment from the HD-MEA chip, while insufficient thickness can make the PDMS difficult to handle.

**Attention:** Be aware that the used Petri dish will be heated to 70°C; the dish should be thermally stable at this temperature to prevent deformation.

5. Repeat step 3 to eliminate any trapped air between the PDMS and the surface of the master mold.

***Note:*** In case air bubbles remain, use air pressure to remove them or repeat the vacuum degassing process. Due to the high viscosity of uncured PDMS, bubbles may take time to rise naturally and escape from the volume.

6. Cure the PDMS by placing the Petri dish containing the PDMS-covered master mold in an oven at 70°C for 1 h ±10 min (Figure 2C).

***Note:*** Verify that the PDMS is cured and solidified, and that it is not sticky, i.e., it does not adhere when touched with a sterile Pasteur pipette.

***Note:*** Depending on the temperature, PDMS can be cured faster at higher temperatures or slower at lower temperatures. It is recommended to consult the manufacturer data sheet. Generally, PDMS can be cured at 60°C–70°C for 1–2 h.

7. After cooling down, use a sterile scalpel to dice out the desired pattern, shape it in the desired form and place it in a new Petri dish (Figure 2D). Use a microscope to guide cutting (problem 2).

**Attention:** Do not apply excessive pressure with the scalpel to prevent damaging or breaking the resin master mold.

8. To finalize the PDMS cast, punch micro-holes (*modules*) of the desired diameter to allow access for cell placement. In this protocol, four circular cavities with Ø2 mm in a 2×2 grid were punched into the PDMS.

***Note:*** These punched cavities will enable cell seeding within the defined microstructure (Figure 2E).

***Note:*** The size of the punched modules should be adapted according to the PDMS design and use. For the specific illustrative example presented here, the casted PDMS device contained a series of parallel microchannels 10 μm wide, 10 μm deep and 400 μm long, so that the punched modules were effectually connected through them (Figures 1B and 2D).

9. Clean the PDMS device to prevent future contamination. For that, immerse the PDMS in 70% ethanol for 1 h.
10. After rinsing 3 times with sterile dH_2_O, the PDMS device is placed facing the channel side up into a new Petri dish for 30 min under ultraviolet germicidal irradiation.

**CRITICAL:** This step is essential, since PDMS can leach organic solvents into the culture medium. Thorough washing is therefore crucial to remove these solvents and minimize undesirable effects on cell viability and experimental results.^27^

11. Finally, let the PDMS device dry for another 1–2 h in sterile conditions. When completely dry, the PDMS is ready to be attached to the pre-cleaned and pre-coated HD-MEA chip.

**Pause point:** Here the protocol can be paused and the PDMS can be stored in a sterile container until usage.

### HD-MEA chip cleaning

**Timing: 1–2 days**

To prevent bacterial and fungal contamination, minimize contact with the well area of the HD-MEA chip and handle the chip only by its edges. Additionally, place the HD-MEA chip in a Petri dish for subsequent handling to reduce direct contact and lower the risk of further contamination.

12. Fill the HD-MEA wells with sterile DPBS to reduce the hydrophobic nature of the CMOS electrode area, and incubate for 20–24 h at 37°C.

***Alternatives:*** Use 1% tergazyme solution (10 g/L) in sterile dH_2_O instead of DPBS. Fill the wells with tergazyme and incubate for 24 h at 37°C in the incubator. Afterwards, remove tergazyme and rinse the wells three times with sterile dH_2_O.

13. After removing the DPBS, load the wells to the edge with 5% Extran and gently clean with a soft brush. Afterwards, clean the whole chip with 5% Extran, except the contact pads.

***Note:*** Prevent any liquid from touching the gold contact pads (Figure 1A) that interface the HD-MEA with external electronics. Keep the pads dry to prevent oxidation.

14. Clean the chip’s wells from residual Extran by repeating step 13 with sterile dH_2_O instead of Extran.
15. Use a stream of air or nitrogen to dry the chips.
16. Transfer the chips into a laminar flow hood. Use a tissue soaked in 70% ethanol to clean the chip surface and contact pads, avoiding the well area, and then place the chip inside a sterile Petri dish.
17. Load the chip’s wells to the edge with 70% ethanol and incubate for 1 h. Afterwards, rinse 3 times with sterile dH_2_O.
18. Sterilize the chips for 30 min under ultraviolet germicidal irradiation. The chips are ready for coating and subsequent PDMS attachment.

**Pause point:** At this step the protocol can be paused. HD-MEA chips can be stored in a sterile environment until usage.

### HD-MEA chip coating

**Timing: 2 days**

To promote cell adhesion and prepare the CMOS electrode area for optimal culture conditions, the chip surface must be coated appropriately. The coating protocol may vary depending on the cell type and experimental requirements. In this protocol, we use rat cortical primary neuronal cultures. Therefore, we apply first a synthetic coating with PLO, allow it to dry, and then attach the PDMS structure. Then, after positioning the PDMS on top of the HD-MEA chip, we apply a laminin coating before seeding the cells. Alternative adhesion-promoting (bio)chemicals may also be used, including human laminin, poly-D/L-lysine (PDL or PLL), polyornithine, polyethyleneimine (PEI), or a combination of these.

**Figure 3:**
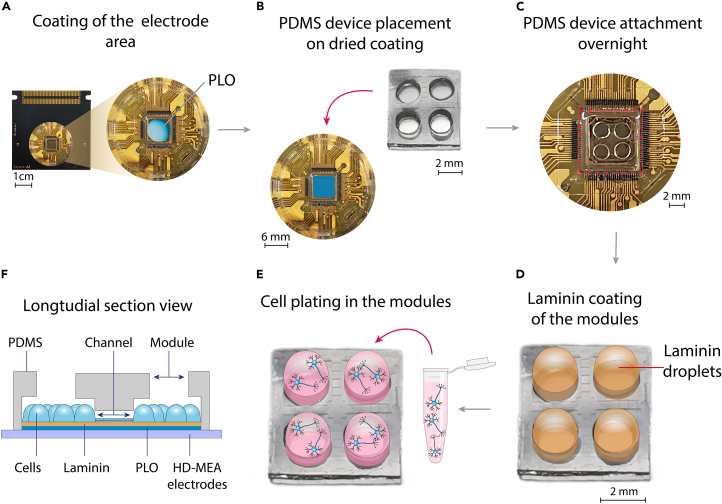
Workflow depicting the manipulation steps from HD-MEA coating to PDMS device placement and cell plating (A) PLO coating of the pre-cleaned recording electrode area in the HD-MEA well. (B) Placement of the PDMS cast on top of the electrode area after washing and drying the well. (C) Improvement of PDMS attachment for 20–24 h. (D) Coating of the electrodes within the modules through laminin droplets. (E) Seeding of the cell culture suspension droplets exclusively on the modules. (F) Illustrative representation of a cross-section of the PDMS-HD-MEA system, showing the plated cells and their interconnectivity throughout the PDMS channels.

19. Coat only the growth area within the chip’s well with 100 μL PLO (50 μg/ml) diluted in DPBS (Figure 3A).

***Note:*** Do not use volumes smaller than 100 μL, as they may evaporate during the incubation period. Place a small container (e.g., a 15 mL falcon lid) filled with sterile dH_2_O and the HD-MEA chip in a big Petri dish to prevent the coating solution from drying out.

20. To ensure that coating covers the complete area, use a pre-cleaned vacuum chamber and apply a vacuum pressure of 60 Torr for 15 min.
21. Incubate the chip containing the coated well for 12 h at 20°C–25°C.
22. Wash 3 times with sterile dH_2_O and let the chip dry out for 3–4 h.

***Note:*** During the first washing step, use a vacuum chamber and apply a pressure of 60 Torr for 10 min to ensure complete removal of unbound PLO.

### PDMS cast placement

**Timing: 2 days**

23. Gently place the PDMS cast with its channel motifs facing down in contact with the desired electrode area of the chip (Figure 3B).

***Note:*** Before placing the PDMS, use a microscope to verify that the microchannels of the PDMS are correctly oriented, i.e., that the microchannels are facing down. Ensure that both the PDMS device and HD-MEA are completely dry to prevent future detachment.

**CRITICAL:** Once the PDMS is placed on the electrodes, it must not be moved or repositioned, as this can damage both the recording electrodes and the coating. Additionally, avoid touching the electrodes with tweezers during PDMS handling, and ensure that the PDMS is placed gently onto the chip (problem 3).

**CRITICAL:** Do not attempt to readjust the position of the PDMS, as this may result in its detachment from the HD-MEA chip.

24. Let the PDMS firmly bond the chip for 20–24 h at 20°C–25°C (Figure 3C).

### Second HD-MEA chip coating

**Timing: 2 days**

Neurons and their processes will grow within the device, adhering to the exposed HD-MEA surface of the punched modules and microchannels. To promote neuronal network development, we apply an additional and essential coating of mouse laminin at a concentration of 10 μg/mL to these surfaces. For details on laminin handling and storage, please refer to the specifications in the materials and equipment section.

25. Apply the coating exclusively to the punched modules of the PDMS. In this protocol, with modules measuring 2 mm in diameter, use 5–10 μL of coating solution per module (Figure 3D).

***Note:*** Adjust the coating volume based on the size of the punched modules.

***Note:*** To prevent the growth of neurites on top of the PDMS during the development of the neuronal network, be sure that the coating solution is placed only inside the punched modules.

26. Place the PDMS-chip device in a vacuum chamber and apply a pressure of 60 Torr for 5–10 min.

***Note:*** This will ensure that the coating solution flows through the microchannels despite the hydrophobicity of PDMS.

27. Incubate at 37°C for 1–2 h and 95% humidity.

**CRITICAL:** Although laminin is often applied with PDL to promote cell attachment, high concentrations or denaturation of PDL —such as when the coating dries before cell seeding— can induce cytotoxic effects and reduce cell viability in culture.

***Note:*** Due to the small volume of punched modules, monitor for evaporation every 10 min, and refill as needed. If a high evaporation rate is observed, place the HD-MEA with a container (e.g. falcon lid) of water in a big Petri dish to increase humidity.

***Note:*** If the laminin dries out, exclude the HD-MEA from further processing.

***Note:*** While incubating, prepare the cell culture for plating the cells onto the chip.

### Culturing cells atop the PDMS-HD-MEA device

**Timing: 2–3 h**

Depending on the cell model and the size of the punched modules, adjust both the cell density and the volume used accordingly.

28. To seed the cells, remove the coating and immediately add 5–10 μL of cell suspension with a density of 2,000 cells/mm^2^ in each punched module on the PDMS (Figure 3E) (problem 5).

***Note:*** To prevent cells from growing on top of the PDMS surface, seed them only inside the punched modules. Micropipettes can be used to guide the media directly into the surface of the cavities.

**CRITICAL:** It is essential to quickly add the cell suspension after removing the coating to prevent the laminin layer from drying, as dehydrated laminin can result in poor cell culture performance.^28^

29. Let cells attach for 1 h at 37°C, 5% CO_2_, and 95% humidity in the incubator.

***Note:*** Since the medium volume is low, we recommend verifying every 10 min that PDMS modules are hydrated. If necessary, gently refill the modules with cell medium to prevent drying out. Additionally, place a container filled with sterile water next to the HD-MEA in a big Petri dish.

30. Depending on the PDMS surface size, **slowly** add 50–100 μL of cell medium on top of the PDMS and incubate for another 1 h in the incubator.

**CRITICAL:** Add the medium very slowly and gently to the cells, as rapid addition can cause cell detachment.

31. To complete the procedure, **slowly** add 1.5 mL of cell medium to the well and place it in the incubator at 37°C, 5% CO_2_, and 95% humidity. Replace half of the medium 2–3 times per week.

***Note:*** When changing the medium, always leave a small volume to ensure the PDMS remains covered with liquid, preventing cell dehydration.

### Recording neuronal activity on PDMS-HD-MEA chips

**Timing: 1 h**

The HD-MEA technology enables recording of neuronal activity within a modular neuronal network under sterile and non-invasive conditions, allowing repeated measurements of the same culture throughout development. Recordings typically begin at day *in vitro* (DIV) 6–7, with neuronal activity monitored for 5–20 min per session, depending on the experimental goal. The HD-MEA system includes an integrated heating unit for temperature control during measurements. Since the HD-MEA CMOS electrodes are highly light-sensitive, the chip must be covered with a dark lid during operation. This lid usually functions as a small chamber that contains an opening for connecting a tube to a CO_2_ supply, helping maintain incubator-like conditions during data acquisition.

32. Place the PDMS-HD-MEA chip on its designated slot in the recording system. Cover the chip with the dark lid and connect the CO_2_ supply.
33. Allow the chip to equilibrate for 10 min at 34°C–37°C and at 5% CO_2_ levels, to let the cells adapt.
34. Record neuronal activity for 5–20 min, depending on experimental goal.

***Note:*** Cells can be recorded for up to 1 h in the system.

**CRITICAL:** Do not record immediately after a medium change. Medium should be replaced either the day before or after the recording session, as fresh medium can affect the spatiotemporal dynamics of neuronal activity.

35. After the recording session, return the chip to the incubator until the next use.

***Note:*** Use the HD-MEA software to extract the spike data and analyze it either with built-in or custom-made tools. Typical acquisition and data analysis parameters for the 3Brain system are: sampling rate of 20 kHz, intrinsic noise level of 11 μV_rms_, 800 Hz high-pass Butterworth filter to remove low-frequency artifacts, and a spikes’ detection algorithm based on ‘Precise timing Spike Detection’ (PTSD)^29^ with a threshold of 5 times the noise level. Typical analysis parameters for MaxOne+ are: sampling rate of 20 kHz, intrinsic noise level of 2.3 μV_rms_, band-pass filter in the range 300–3,000 Hz to remove low-frequency artifacts, and automatic spike detection considering a threshold 5 times the noise level.^1^ Details on data analysis in HD-MEA data and the impact of parameters are discussed in the recent work by Wolff et al.^30^

### Cleaning the HD-MEA chip after usage

**Timing: 30 min**

36. Carefully remove the PDMS from the HD-MEA chip using tweezers, avoiding contact with the electrodes.
37. Fill each chip well to its edge with 5% Extran solution and gently clean it using a soft brush. Incubate for 30 min.
38. Additionally, clean the chip surface with the 5% Extran, avoiding the gold contact pads.

***Note:*** Do not soak the contact pads with liquid.

39. Wash the well and the chip surface twice with sterile dH_2_O to completely remove any detergent residue.
40. Dry the chip by using a stream of air or nitrogen. Store the chip in a sterile environment until the next use (problem 4).

## Expected outcomes

The presented protocol outlines the generation of tailored *in vitro* neuronal cultures with modular characteristics, providing a platform for the formation of compartmentalized neuronal circuits and enabling the study of activity and functional connectivity under defined conditions. For both 3Brain and MaxWell systems, neuronal activity typically emerges in the considered rat cortical cultures around DIV 6-7, and recordings can be performed daily on the same culture up to DIV 21. Within this period, cultures remain consistently active and exhibit synchronous, network-wide events starting at approximately DIV 10. After each recording session, data are processed using built-in software to extract neuronal spike trains from electrode-level signals. This data is stored in plain text files for subsequent analysis, such as activity quantification or effective connectivity. The influence of the modular design on activity is evident from the first recording day, with neurons initially activating sparsely within individual modules and progressively establishing interactions with other modules as intra-module connections mature. In the following sections, we first present results obtained using the 3Brain HD-MEA platform to illustrate the impact of modular constraints on neuronal activity. We then demonstrate the implementation of the protocol on the MaxWell Biosystems platform, highlighting the reproducibility and the cross-platform adaptability, and the preservation of key network features. Finally, we include scanning electron microscopy (SEM) images of the HD-MEA substrates from the two different providers, to offer a comparative structural characterization of the electrode surface topographies.

**Figure 4:**
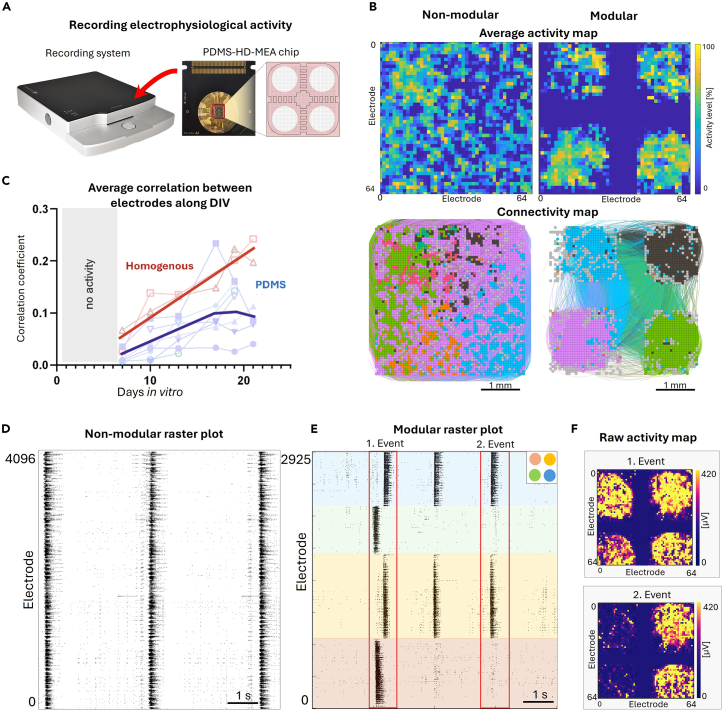
Results comparing neuronal dynamics in non-modular (homogeneous) and modular (PDMS-implemented) cortical networks grown on a 3Brain system (A) Illustration of the recording system and the PDMS-HD-MEA chip. (B) Top: average activity map at day in vitro (DIV) 14 illustrating the confinement of activity within segregated areas for the modular configuration. Color brightness is proportional to the degree of activity in the electrodes. Bottom, corresponding effective connectivity maps, showing the capacity of the modules to interact to one another via the built-in channels in the PDMS devices. Colors highlight groups of electrodes that strongly interact, which more prominently occur within the modules. (C) Average correlation between active electrodes in the homogeneous and modular networks during development, from DIV 7 to 21. Symbols indicate experimental repetitions, and thick lines show the general average trend for each experimental condition. (D) Raster plot of spontaneous activity at DIV 14 in the full chip for a homogeneous network. Black dots are neuronal activations. (E) Equivalent data for a modular network. Colors bands indicate the specific modules according to the color scheme provided in the top-right corner of the panel. Red boxes highlight two contrasting collective events, one integrated (event 1) and one segregated (2). (F) Corresponding activity maps of the collective events 1 and 2.

To demonstrate the impact of structural network architecture on neuronal dynamics, we compared modular and non-modular networks grown on HD-MEA chips using the 3Brain platform (Figure 4A). Non-modular neuronal cultures displayed uniformly distributed activity due to the formation of random connectivity. In contrast, modular networks showed localized activity confined to the PDMS-defined modular regions (Figure 4B). Analysis of network connectivity revealed that the non-modular network formed widespread interconnections, while the modular network displayed dense intra-module interconnectivity combined with specific inter-module connections (Figure 4D). Furthermore, neuronal activity was monitored during development from early (7 DIV) to late developmental stages (21 DIV) to capture the evolution of network dynamics during maturation. Non-modular networks exhibited a progressive increase in average correlation of neuronal activity patterns over time, indicating growing synchrony. Modular networks, however, displayed a more varied activity regimes throughout development, suggesting that the defined modularity supports stable segregation of activity over time (Figure 4C). Indeed, raster plots revealed that non-modular networks developed highly synchronized collective bursts events, whereas modular networks exhibited more diverse spatiotemporal dynamics. These included synchronized collective bursts within individual modules and coordinated activation across modules, revealing the capacity of modular networks to support both module-level activity (segregation) and network-wide coactivation (integration) (Figures 4E and 4F). Two representative events, highlighted as red boxes in the raster plot of Figure 4F, are presented. In the first event, all modules activated sequentially within a time window (integration, Figure 4G top), whereas in the second event, only two modules were activated together (segregation, Figure 4G bottom). Taken together, these results demonstrate that PDMS microstructures effectively constrain network growth and connectivity in a distinct, defined modular organization. This structural modularity is preserved over time and supports enriched, complex dynamics that depart from the rigid behavior of the non-modular design.

**Figure 5:**
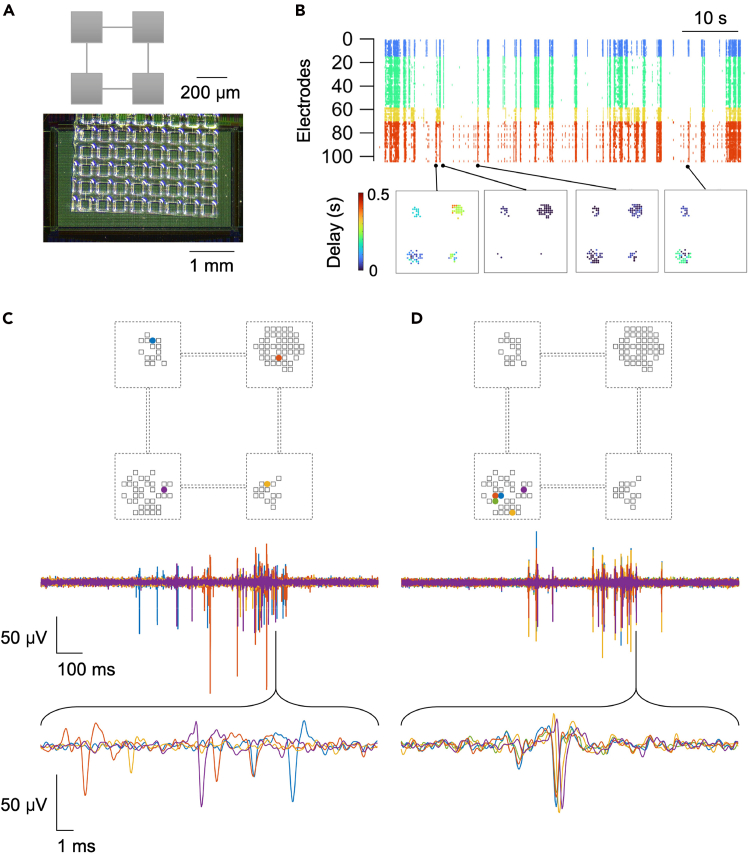
Representative results of spontaneous activity in modular cultures grown in the MaxOne system (A) Schematic Illustration of the modular network (top) and the microfabricated PDMS placed in the HD-MEA (bottom). (B) Top: Neuronal activity of a modular network plotted over time as a raster plot; electrodes belonging to different modules are color-coded. Bottom: corresponding propagation of neuronal activity, represented at different time points. (C and D) Activity measured in different modules and within one module (top), while the corresponding-colored waveforms are displayed underneath. Figure reprinted and adapted with permission from Sato et al.^1^

**Figure 6:**
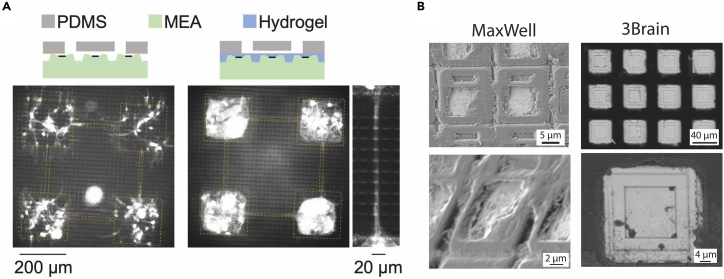
Overview of the topographical surface of the electrode area of HD-MEA chips (A) Integration of the PDMS microstructure on the MaxWell HD-MEA chip, using a fluorescence dye (NeuO) to stain beta-III-tubulin to show growth of neurites without (left) and with (right) a hydrogel coating. The right panel shows a detail of a microchannel connecting two modules. Reproduced with permission from Sato et al.^1^ (B) SEM images of the electrode area of the HD-MEA chip from MaxWell (without hydrogel coating, left) and from 3Brain (right).

The applicability of PDMS-based cell patterning to HD-MEAs from other manufacturers is demonstrated in Figures 5 and 6. Here, we employed the MaxOne HD-MEA chip from MaxWell Biosystems, and the microstructured PDMS device was prepared to allow the design of smaller networks. The micro-holes in the PDMS now measure 200 μm per side (Figure 5A), in contrast to the 2 mm holes used in previous data. The fabrication process of these devices has been detailed in Murota et al.^31^. Similar to the larger modular networks shown earlier (Figure 4E, right), these micropatterned neuronal networks exhibited complex spontaneous activity, characterized by the coexistence of segregated and integrated activity patterns, even at this reduced scale (Figure 5B). During episodes of globally synchronized bursting, activity propagated across modules over a timescale ranging from several milliseconds to several tens of milliseconds (Figure 5C). We note that, while propagation occurring over tens of milliseconds can be resolved with calcium imaging,^32^ that on the millisecond timescale is typically challenging to capture due to the temporal limitations of the imaging devices and the kinetic properties of calcium indicators; hence, the need for HD-MEA. The sub-millisecond sampling rate of HD-MEAs even allows us to resolve activity propagation within single modules (Figure 5D). Detailed information for these specific experiments is provided in Sato et al.^1^ Due to the microtopographical features on the surface of the original MaxOne chip, additional processing steps (hydrogel coating to flatten the surface) were required to attach PDMS devices with no observed reduction in electrode sensitivity (Figure 6A).^1^^,^^13^

SEM imaging highlights substantial differences in electrode surface topography between HD-MEA providers, which can critically influence the PDMS adhesion and the neurite growth patterns. Specifically, 3Brain chips exhibit recessed electrode areas, whereas MaxWell chips display elevated electrode structures (Figure 6B). Such variations should be carefully considered when designing experiments, as they can affect the stability of the physical interface and the guidance of the neurite outgrowth. However, with the newer MaxOne+ chip with a flatter surface, PDMS devices can be placed directly onto it without any non-specific neurite outgrowth beneath the PDMS, and thus its use is highly recommended. We have so far only employed the MaxOne+ chips with polyethyleneimine/laminin coating, but, in principle, the PLO/laminin coating described here for 3Brain chips should also be applicable.

Taken together, these results provide an overview of the compatibility of our modular network protocol across different HD-MEA platforms and their spatial configurations. This cross-platform adaptability supports broader applications in the investigation of neuronal dynamics, synaptic integration, network computation, and neuronal development.

## Limitations

### Limitation 1: PDMS design

The electrode area and the layout vary within different HD-MEA chips, depending on the chip type and manufacturer. Small areas may substantially determine the design and final size of the PDMS structure. Thus, the variability of the design within different HD-MEA chips constrains the pattern and dimensions of the PDMS structure, which must be precisely aligned with the electrode area. Consequently, the protocol may require adaptation when applied to different HD-MEA chips (microfabricated device design).

### Potential solution

When planning the PDMS cast design, verify the exact sizes of the growth and electrode area of the HD-MEA chip. For the CorePlate™ 1W 38/60 (3Brain), the size must be smaller than the full growth area (6 ×6 mm^2^) to fit inside the well, and the design itself should approximately fit the size of the electrode area (3.8 ×3.8 mm^2^) to assure the cells grow on top of the electrodes (Figure 1). For the MaxOne (MaxWell), be aware that the electrode area is 3.85×2.10 mm^2^ (Figure 5A).

### Limitation 2: HD-MEA topography variation within manufacturers

Depending on the specific chip model, the electrode surface topography is often not entirely flat, which impacts the good contact and attachment of PDMS on the chip’s surface and may affect the reproducibility of the data as well as the expected results in terms of constrained neuronal connectivity. 3Brain chips have electrodes that are recessed, whereas MaxOne+ chips have electrodes that are elevated (Figure 6B).

### Potential solution

When planning the experimental setup, inspect the topography of the chip under a microscope or request chip information from the provider. When working with non-planar surfaces, a thin intermediate biolayer coating, such as collagen, may be used to improve surface uniformity (Figure 6A).^1^

### Limitation 3: Non-transparency of HD-MEAs

Due to the complex, multilayered electronic architecture, most of the HD-MEA chip materials are non-transparent, making it challenging to monitor neuronal morphology and development when culturing cells on them (culturing cells atop the PDMS-HD-MEA device).

### Potential solution

To overcome this limitation, we recommend using live fluorescence dyes such as NeuO, which labels beta-III-tubulin, to visualize neurite growth in combination with an upright microscope (Figure 6A).^1^ Additionally, it is recommended culturing neurons in parallel on a transparent surface, such as a glass coverslip, to monitor cell morphology and development over time, providing complementary insights into network behavior. Neuronal activity on these transparent surfaces can be tracked using calcium imaging.

### Limitation 4: Batch-to-batch variability in PDMS curing

Variations in mixing accuracy, curing temperature, and reagent age can alter PDMS stiffness and shrinkage, potentially affecting feature conformity (PDMS preparation and cleaning).

### Potential solution

This is minimized by consistent mixing ratios, controlled curing conditions, and fresh reagents. For features >1 μm, these effects are generally negligible and do not compromise pattern fidelity.

### Limitation 5: Variability in SU-8 master height

Spin speed, humidity, temperature, and soft-bake uniformity can introduce ±5–10% variation in SU-8 thickness (master inverse device generation).

### Potential solution

Profilometry or microscopy checks, together with routine recalibration of coating parameters, maintain reproducibility across master batches.

### Limitation 6: Long-term PDMS warping

PDMS may undergo slow mechanical relaxation during extended culture periods, causing minor warping or dimensional drift (PDMS preparation and cleaning).

### Potential solution

To prevent these effects, PDMS devices should be used within a defined timeframe, ideally within 12 months of fabrication. It is also recommended to use only freshly fabricated PDMS devices and avoid reusing them, as any material deformation accumulated over time may be transferred to the new neuronal culture.

### Limitation 7: HD-MEA electrode aging

Repeated sterilization and prolonged use can degrade MEA electrodes, increasing impedance and reducing signal quality (HD-MEA chip cleaning).

### Potential solution

Routine impedance monitoring and periodic electrode replacement are recommended to maintain stable electrophysiological performance. Once the HD-MEA chip reaches the end of its functional lifetime, it should be replaced with a new one.

## Troubleshooting

### Problem 1: Risk of contamination

The non-sterile and adhesive properties of PDMS represent a high risk of contamination for the HD-MEA chips, thereby compromising their intended reusability (PDMS preparation and cleaning).

### Potential solution

To prevent contamination, use only sterile materials when handling PDMS. Furthermore, it is recommended to perform the PDMS preparation in a Class 10.000 cleanroom, which is optimized for fabrication of microstructures and minimizes aerosolized contamination that could compromise the cleanliness of the HD-MEA chips. Alternatively, PDMS devices can be sterilized under UV light inside a biological safety cabinet. It is worth noting that PDMS can absorb some solvents. Thus, although sterilization using ethanol is often effective, the PDMS devices should be soaked in buffer or culture media for several few hours before cell seeding to ensure complete removal of residual ethanol.

### Problem 2: Reproducibility of the punched micro-holes

The final design of the PDMS cast needs to be tailored to the electrode area of the HD-MEA chip. Placement and alignment of the PDMS is normally carried out by hand, which is challenging due to the small scale of the design, especially when punching the micro-holes that allow cell access to the chip surface. Thus, achieving consistent spacing between these holes is difficult, which can lead to variability in the spatial arrangement of compartments. In turn, this may affect the length of crossing neurites between two modules and their overall interconnectivity, ultimately reducing the reproducibility of the experiments (Step 8 in PDMS preparation and cleaning).

### Potential solution

To improve the reproducibility and precision of the micro-holes, it is recommended to use a punching system with an integrated camera, such as the PDMS Port Creator (CorSolutions). Alternately, a microscope could assist in accurately positioning the puncher. Visual guidance increases precision during punching and thus the consistent placement of the modules.

### Problem 3: Increased noise level during the recordings in the PDMS-bonded areas

Prolonged presence of PDMS over the electrodes often leads to a substantial increase in noise levels, which can negatively affect the final quality of the recordings and data accuracy (PDMS cast placement).

### Potential solution

Electrodes in direct contact with the PDMS should be excluded from both the recording and subsequent analysis. If possible, a brightfield image of the electrode area can be overlaid with the electrode map, thus allowing to manually select and exclude the affected electrodes. Additionally, it is critical to control the recordings and re-evaluate the recorded activity data. In case PDMS-induced noise is detected, software-based filtering should be used to identify and remove noisy electrodes.

### Problem 4: PDMS residues

After PDMS removal and cleaning, residues may remain on some electrodes, which can lead to a negative impact on future recordings (cleaning the HD-MEA chip after usage).

### Potential solution

To remove residual PDMS on the HD-MEA chip after use, and to ensure clean electrodes for subsequent preparations, incubate the HD-MEA chip for a minimum of 2 h to a maximum of 24h with tergazyme. Due to the hydrophobic nature of PDMS, residues may trap air bubbles, which can interfere with recordings. Thus, after incubation, connect the chip with the HD-MEA system and assess electrodes activity. If elevated noise levels are observed, suggesting that air bubbles are still present, repeat the chip-cleaning process using Extran or tergazyme detergents (cleaning the HD-MEA chip after usage).

### Problem 5: Cell seeding

Plating cells in the micro-holes can be challenging due to the difficulty of accurately placing small volumes of cell suspension without touching the PDMS. Accidental contact may cause the PDMS to detach from the HD-MEA chip. Additionally, the small volume of cell suspension tends to evaporate fast, which can hinder cell attachment and lead to an uneven cell distribution or even complete cell loss (culturing cells atop the PDMS-HD-MEA device).

### Potential solution

To improve handling and minimize delays during cell plating, it is recommended to practice the placement of small volumes of cell suspension into the micro-holes using unused PDMS casts prior the actual experiment. This leads to faster and more efficient plating and ultimately reduces the time the suspension is exposed to air, minimizing evaporation. An alternative approach is to apply a larger drop of cell suspension that covers all micro-holes simultaneously. However, this method may lead to unwanted cell attachment and growth on the PDMS surface between the micro-holes. To mitigate this, coating the PDMS surface with agarose prior to cell seeding can help prevent nonspecific cell adhesion.

## Resource availability

### Lead contact

Further information and requests for resources should be directed to the lead contact, Jordi Soriano (jordi.soriano@ub.edu).

### Technical contact

Technical questions on executing this protocol should be directed to and will be answered by the technical contact, Anna-Christina Haeb (haeb.anna@web.de), Hideaki Yamamoto (hideaki.yamamoto.e3@tohoku.ac.jp), and Paul Roach (p.roach@lboro.ac.uk).

### Materials availability

This study did not generate new or unique reagents.

### Data and code availability

This study did not generate new data or unique codes.

## Acknowledgments

The work was partly supported by 10.13039/501100001700MEXT Grant-in-Aid for Transformative Research Areas (A) “Multicellular Neurobiocomputing” (24H02332 and 24H02333), 10.13039/501100001691JSPS KAKENHI (22KK0177 and 23H03489), and the Cooperative Research Project Program of the RIEC, Tohoku University. This research was partly carried out at the Laboratory for Nanoelectronics and Spintronics, RIEC, Tohoku University. This research was supported by the 10.13039/100011103European Union Horizon 2020 research and innovation program under grant no. 964877 (project NEU-CHiP). The work was partly supported by the 10.13039/100014440Ministerio de Ciencia, Innovación y Universidades (Spain), under projects PID2022-137713NB-C22 (J.S.), PID2023-146800OB-I00 (D.T.), and CNS2023-143862 (D.T.); and 10.13039/501100010552Generalitat de Catalunya (Spain) under the projects 2021-SGR-01086 (D.T.) and 2021-SGR-00450 (J.S.).

## Author contributions

A.-C.H., Y.S., J.S., D.T., and H.Y. conceived of the experiments and framework. D.M., A.-C.H., P.R., Y.S., D.T., J.S., and H.Y. designed the patterns. D.M. and Y.S. fabricated the master molds. A.-C.H. planned and carried out the PDMS protocol, initial cell culturing, and data analysis for the 3Brain system, and Y.S. carried out the initial cell culturing and data analysis for the MaxWell system. D.M. and P.R. carried out the SEM experiments. A.-C.H. wrote the initial draft. J.S., H.Y., D.T., Y.S., D.M., and P.R. edited and reviewed the manuscript.

## Declaration of interests

The authors declare no competing interests.
